# N-Terminal T4 Lysozyme Fusion Facilitates Crystallization of a G Protein Coupled Receptor

**DOI:** 10.1371/journal.pone.0046039

**Published:** 2012-10-04

**Authors:** Yaozhong Zou, William I. Weis, Brian K. Kobilka

**Affiliations:** 1 Department of Molecular and Cellular Physiology, Stanford University School of Medicine, Stanford, California, United States of America; 2 Department of Structural Biology, Stanford University School of Medicine, Stanford, California, United States of America; Medical School of Hannover, United States of America

## Abstract

A highly crystallizable T4 lysozyme (T4L) was fused to the N-terminus of the β_2_ adrenergic receptor (β_2_AR), a G-protein coupled receptor (GPCR) for catecholamines. We demonstrate that the N-terminal fused T4L is sufficiently rigid relative to the receptor to facilitate crystallogenesis without thermostabilizing mutations or the use of a stabilizing antibody, G protein, or protein fused to the 3rd intracellular loop. This approach adds to the protein engineering strategies that enable crystallographic studies of GPCRs alone or in complex with a signaling partner.

## Introduction

Obtaining well-diffracting crystals of G-protein coupled receptors remains one of the most challenging obstacles for structural studies of this important family of signaling proteins. Only a limited number of GPCR structures have been determined by x-ray crystallography. A factor contributing to the difficulty in obtaining GPCR crystals is the relatively small amount of polar surface area available for forming crystal lattice contacts.

We previously developed two strategies to address this problem. First, a stabilizing antibody was used to facilitate the crystallization of human beta2 adrenergic receptor (β_2_AR) [1], and more recently to stabilize and crystallize the active state of the β_2_AR [2]. These antibodies bind and stabilize the cytoplasmic ends of transmembrane segments (TM) 5 and 6, and provide a structured hydrophilic surface for crystal packing interactions. In the second approach, T4 lysozyme (T4L) was fused to the cytoplasmic ends of TM5 and TM6, replacing the unstructured intracellular loop 3 (ICL3) [3]. The fused T4L formed packing interactions in the crystal lattice and resulted 2.4 Å crystal structure. Importantly, the TM5-T4L-TM6 fusion approach has been effective for at least seven other GPCRs [4], [5], [6], [7], [8], [9], [10].

Although both of the strategies have been effective for crystallizing isolated GPCRs, neither can be used to facilitate crystallization of signaling complexes such as GPCR-G protein and GPCR-arrestin complexes, where the antibody or the fused T4L would interfere with complex formation. We therefore explored the use of T4L insertions on the extracellular surface of the β_2_AR. The extracellular loops of the β_2_AR and other GPCRs do not tolerate large insertions or deletions. In contrast, the amino terminus of the β_2_AR can be deleted without loss of function. We therefore chose to replace the N terminus of the β_2_AR with T4 lysozyme (T4L-GPCR fusion).

## Results

### Fusion of a T4L to the N-terminus of β_2_AR

**Figure 1:**
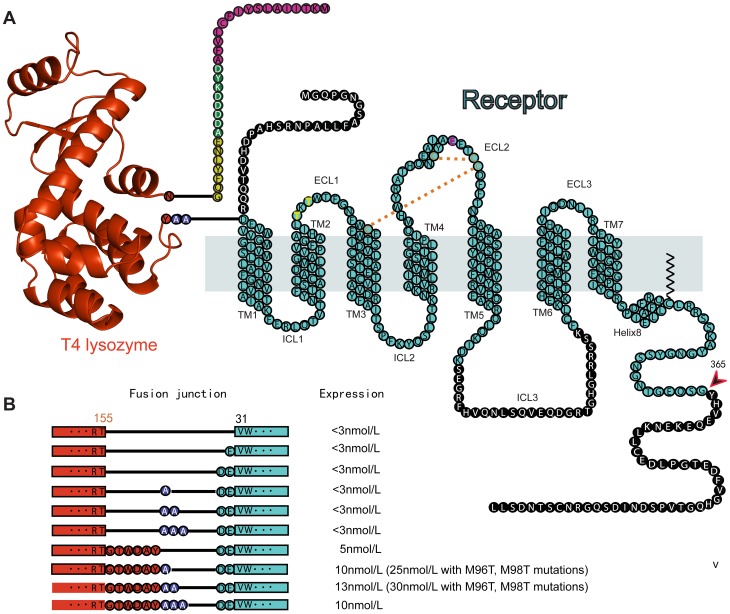
Modifications of the β_2_AR to facilitate crystallogenesis. **A**. Schematic diagram of T4L-β_2_AR-ΔICL3 fusion protein used for crystallography. Cyan circles indicate β_2_AR residues included in the construct. Black circles with white letters indicate the residues deleted from the wild type β_2_AR sequence. Pink circles indicates the HA signal peptide. Green circles indicate the FLAG tag. Yellow circles indicate the TEV recognition site. Cyan circles with yellow letters indicate the M96T, M98T mutations. Cyan circles with red letters indicate the cysteines involved in disulfide bonds. Gold dashed lines indicate the disulfide bond linkages. Cyan circle with a white letter indicates the N187E mutation. Blue circles indicate the 2-Ala linker. Red circles indicate T4L residues. **B**. Schematic diagram of all of the T4L-β_2_AR-ΔICL3 constructs that were generated and evaluated for expression of functional receptor protein in insect cells. Red bar and circles represent T4L sequence. Cyan bar and circles represent β_2_AR. Blue circles represent Ala linkers.

To have a T4L-β_2_AR fusion protein suitable for crystallization, the link between T4L and the receptor must be short and relatively rigid, yet not interfere with receptor function. Several different fusion proteins were generated and examined for expression levels and binding properties (Fig. 1). In an effort to generate a rigid interaction between T4L and the β_2_AR, we removed the relatively flexible C-terminus of the T4L and attempted to fuse the remaining C terminal helix of T4L with the extracellular end of TM1 of the β_2_AR. None of these constructs gave sufficient amounts of functional receptor.

In a second approach, we fused the carboxyl terminus of T4L to D29, the first amino acid of the extracellular helical extension of TM1. Four constructs were generated and examined: direct fusion of T4L to D29, and the inclusion of 1–3 Ala residues between T4L and the β_2_AR (Fig. 1). The highest level of expression was obtained from the fusion with a two-Ala linker. The fusion protein had normal pharmacology and G protein coupling. To improve expression, two additional point mutations M96T and M98T were made in the β_2_AR component of the fusion protein. We have previously observed that mutation of these residues, which are located in the first extracellular loop and face away from the protein, had no effect on receptor function, but enhanced expression by up to two-fold. We were able to produce 1.5mg of pure, functional protein from 1 liter of Sf9 cells (Expression Systems, Woodland, CA).

### The role of the N-T4L in facilitating crystallogenesis

**Figure 2:**
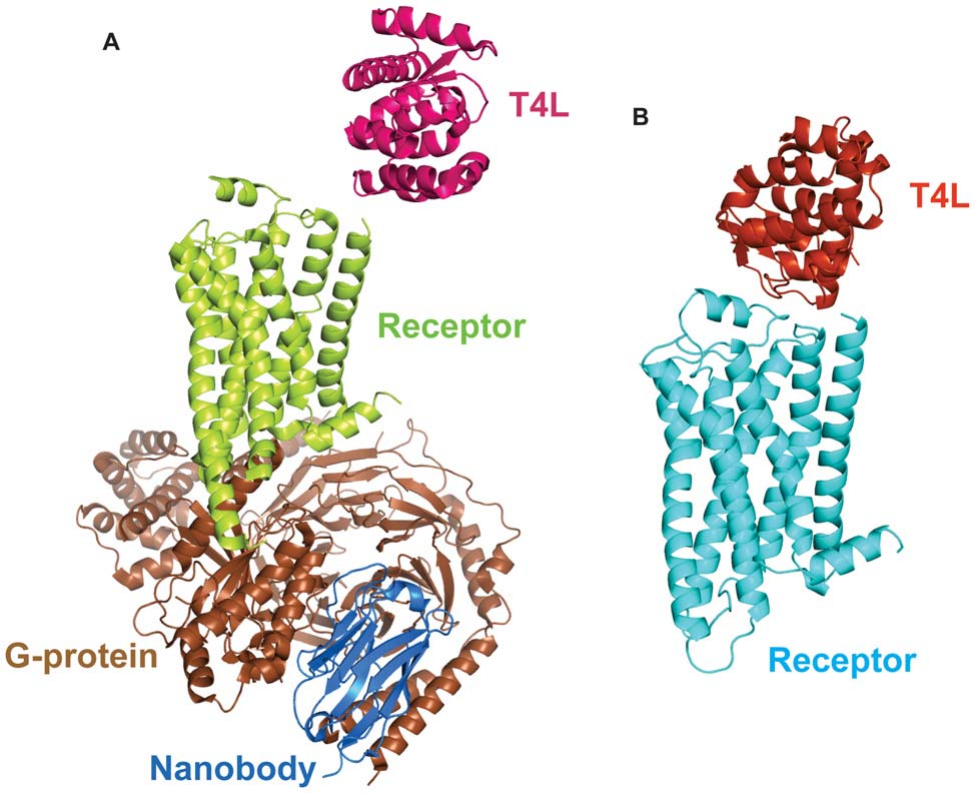
The T4L moiety in different crystal structures. **A.** The crystal structure of the β_2_AR-Gs complex. The T4L is shown in magenta. The β_2_AR is shown in green. The G-protein heterotrimer is shown in brown. The stabilizing nanobody is shown in blue. There are no interactions between the T4L and its fused β_2_AR. **B.** The crystal structure of T4L-β_2_AR-ΔICL3. The T4L is shown in red and its fused β_2_AR-ΔICL3 is shown in cyan. In contrast to the β_2_AR-Gs complex structure, there are interactions between the T4L and its fused receptor β_2_AR-ΔICL3.

The above version of T4L-β_2_AR was recently used to obtain the crystal structure of the β_2_AR-Gs complex [11]. However, in this structure most of the lattice contacts in this crystal are mediated by Gs, and the N terminal fused T4L does not interact with the extracellular surface of its fused β_2_AR (Fig. 2). The lack of interactions between T4L and the extracellular surface of the β_2_AR in the β_2_AR-Gs complex suggested that T4L fused to the N terminus of the β_2_AR might not be sufficiently constrained to facilitate crystallogenesis in the absence of the cytoplasmic G protein. We therefore sought to determine if the amino terminal T4L could facilitate crystallogenesis in the absence of a soluble protein bound or fused to the third intracellular loop. Additional modifications were made to minimize unstructured sequence in the third intracellular loop and carboxyl terminus (Fig. 1). We truncated the C-terminal residues after amino acid 365. The 3^rd^ intracellular loop (ICL3) of β_2_AR is another flexible region and it is subject to proteolysis [1]. This loop was truncated in the fusion protein by removing residues 235 to 263. The final construct T4L-β_2_AR-Δ-ICL3 is illustrated in Figure 1.

**Figure 3:**
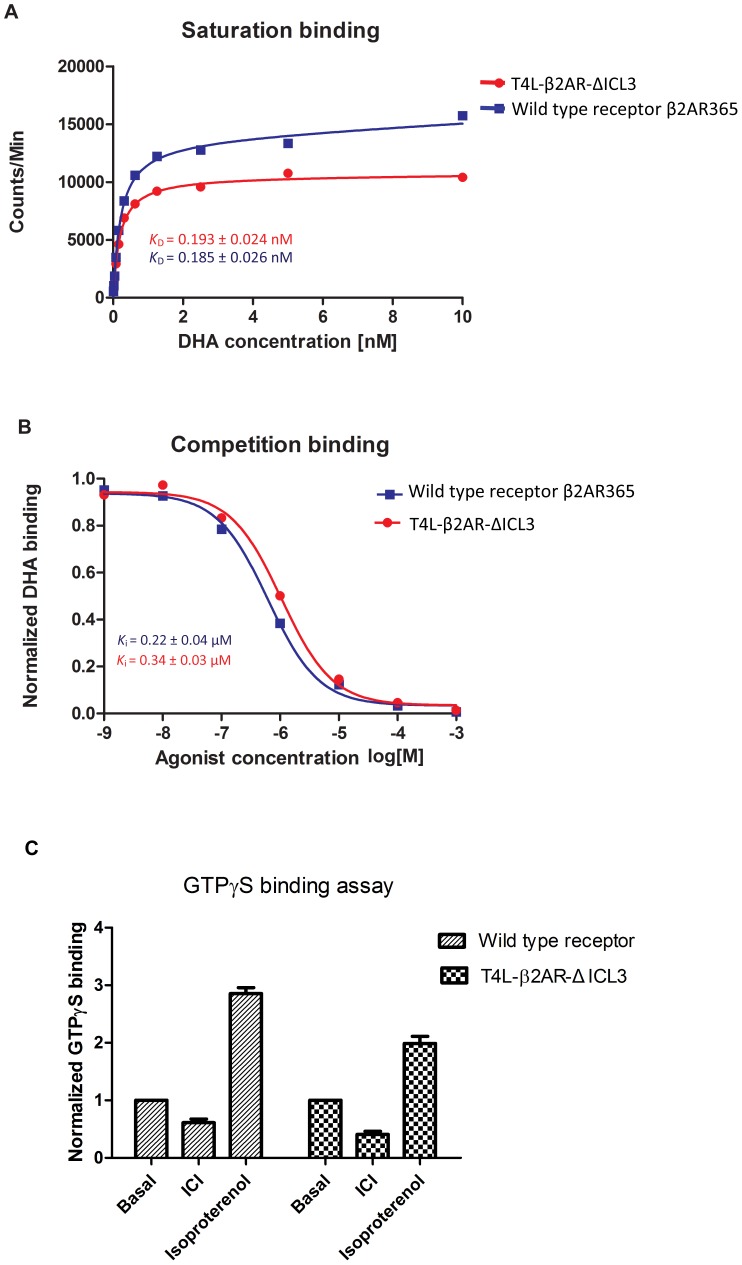
Biochemical characterization of the modified β_2_AR. **A.** Saturation binding analysis for the antagonist [^3^H]dihydroalprenolol (DHA) binding to T4L-β_2_AR-ΔICL3 and the wild type β_2_AR365. **B.** Competition binding analysis for the agonist isopreterenol competing for binding of DHA to T4L-β_2_AR-ΔICL3 and the wild type β_2_AR365. **C.** GTPγS binding to the G protein Gs mediated by T4L-β_2_AR-ΔICL3 and the wild type β_2_AR365 in the presence of different ligands. Receptors and G proteins were reconstituted into HDL particles as described in Materials and Methods.

To determine the functional integrity of T4L-β_2_AR-Δ-ICL3, we determined agonist and antagonist binding affinities. The ligand binding pocket is formed by amino acids from four transmembrane domains and is therefore very sensitive to any perturbation of the receptor structure. T4L-β_2_AR-Δ-ICL3 exhibits ligand binding affinities for the antagonist [3H]-Dihydroalprenolol and the agonist isopreterenol that are comparable to those of the wild type receptor (Fig. 3). T4L-β_2_AR-Δ-ICL3 also maintains the ability to couple to the G-protein Gs (Fig. 3C). The inhibition of basal GTPγS binding by the inverse agonist ICI-118551 is slightly greater for T4L-β_2_AR-Δ-ICL3 than for the wild-type β_2_AR. This observation suggests that the modifications used in constructing T4L-β_2_AR-Δ-ICL3 might lead to constitutive activity; however, the observed difference is not statistically significant and T4L-β_2_AR-Δ-ICL3 does not exhibit higher affinity for agonists (Fig. 3B), a property often associated with constitutive activity in GPCRs.

**Figure 4:**
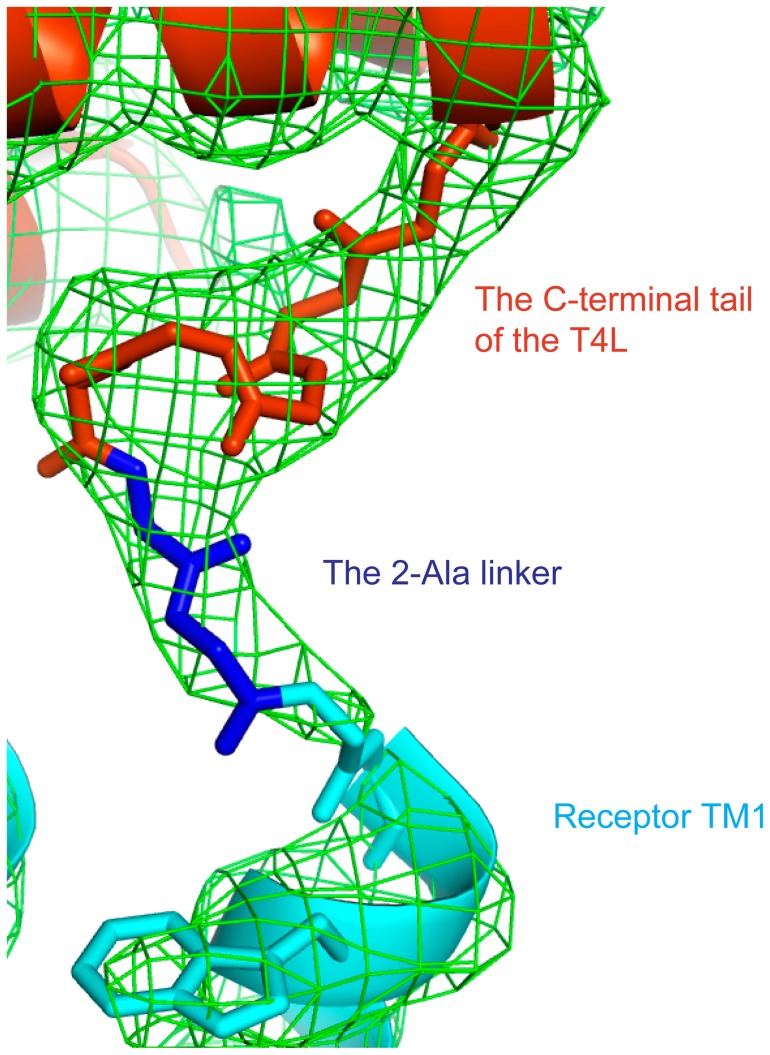
2Fo-Fc omit map around the 2-Ala linker between T4L and the β_2_AR. The T4L residues Gly156-Tyr161, the 2-Ala linker and the receptor residues D29-E30 are not included in the map calculation. The main chain of the fusion junction is shown in sticks. The electron density is shown in green mesh (0.9 σ). The T4L is shown in orange. The β_2_AR-ΔICL3 is shown in cyan. The 2-Ala linker is shown in blue.

**Figure 5:**
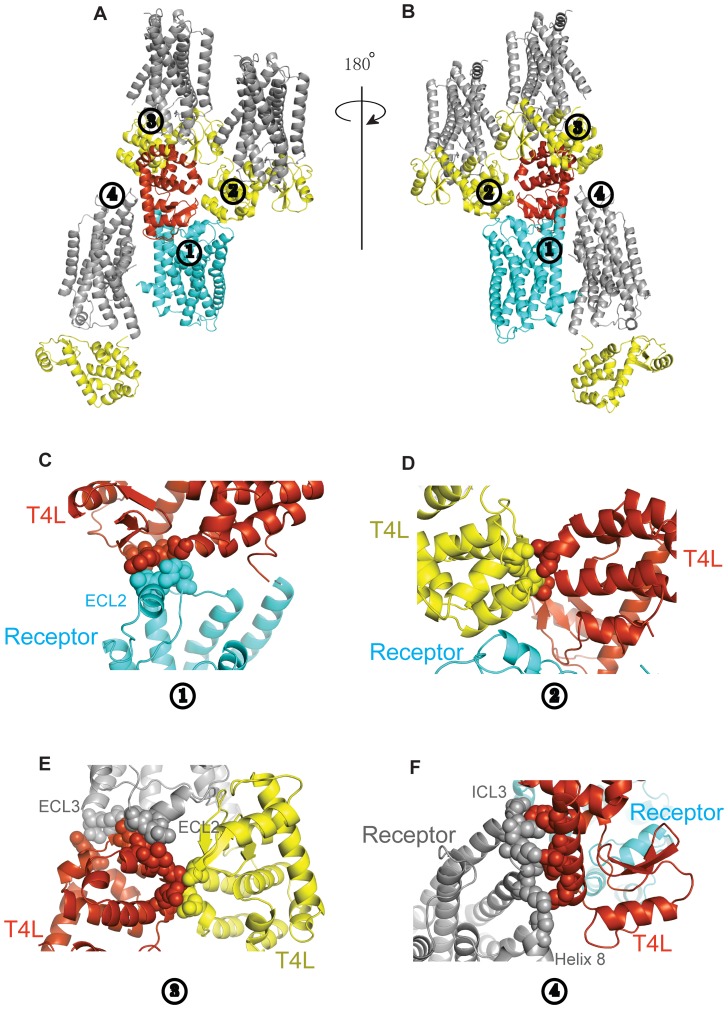
Packing interactions in the crystal structure. **A, B.** Packing interactions mediated by T4L. Each T4L packs against three adjacent T4L-β_2_AR-ΔICL3 molecules and is involved in 4 packing interactions. The T4L and β_2_AR-Δ-ICL3 from the reference molecule are shown in orange and cyan, respectively. The T4L and β_2_AR-Δ-ICL3 from the three adjacent molecules are shown in yellow and dark grey, respectively. **C–F.** Close-up view of packing interactions 1–4. The residues involved in interactions are shown as spheres **C.** In interaction 1 the reference T4L packs against ECL2 of its fused β_2_AR-Δ-ICL3. **D.** In interaction 2 the reference T4L packs against T4L of an adjacent T4L-β_2_AR-Δ-ICL3. **E.** In interaction 3 the reference T4L packs against T4L, ECL2 and ECL3 of a second adjacent T4L-β_2_AR-Δ-ICL3. **F.** In interaction 4 the reference T4L packs against ICL3 and helix 8 of a third T4L-β_2_AR-Δ-ICL3.

Purified T4L-β_2_AR-Δ-ICL3 bound to the inverse agonist carazolol crystallized as small rods in lipid cubic phase (37% PEG300 (v/v), 0.1M Bis-Tris propane, pH 6.5, 0.1 M ammonium phosphate). Crystals diffracted to a resolution of 3.3 Å; however, due to radiation damage, our dataset was limited to 4.0 (Table 1). Nevertheless, the dataset allowed us to solve the structure by molecular replacement. The interaction between the β_2_AR and T4L is sufficiently rigid to detect electron density for the 2 Ala link between these two proteins (Fig. 4). This link was not detectable in the electron density map of the β_2_AR-Gs structure [11] (Fig. 2). In the T4L-β_2_AR-Δ-ICL3 crystal lattice, the packing interactions are primarily mediated by T4L and there are no contacts between adjacent receptors (Fig. 5), indicating the important role of the T4L in facilitating GPCR crystallization. Each T4L has four packing interactions: 1- against ECL1 and ECL2 of its fused β_2_AR-Δ-ICL3, 2- against T4L of one adjacent T4L-β_2_AR-Δ-ICL3, 3- against T4L, ECL2 and ECL3 of a second T4L-β_2_AR-Δ-ICL3, and 4- against ICL3 and Helix 8 of a third T4L-β_2_AR-Δ-ICL3 (Fig. 5).

**Table 1:** Data collection and refinement statistics.

Data collection	
Space group	P2_1_2_1_2_1_
Unit cell dimensions	
*a*, *b*, *c* (Å)	51.4, 71.4, 161.4
Resolution (Å)	50-4.0 (4.07-4.00)*
*R*_merge_	0.199(0.799)
<*I*/σ*I*>	8.4 (1.5)
Completeness (%)	84.3 (71.2)
Multiplicity	4.7 (3.7)

Refinement	
Resolution (Å)	30-3.99
No. reflections work/free	4547/691
*R*_work_/*R*_free_	0.267/0.293
No. atoms	3623
Average B values (Å^2^)	
Receptor	197
T4L	177
Carazolol	160
Overall anisotropic B (Å^2^)	
B11/B22/B33	−21.2/59.3/−38.0
R.m.s deviations	
Bond lengths (Å)	0.004
Bond angles (°)	0.68
Ramachandran plot **	
% favored	96.4
allowed	3.6
generously allowed	0.0
disallowed	0.0

* High resolution shell in parenthesis.

** As defined by Molprobity.

R_merge_ = Σ*_hkl_* Σ*_i_* |I*_i_*−<I>|/Σ*_hki_* Σ*_i_* I*_i_*.

### Comparison of T4L-β_2_AR-Δ-ICL3 and β_2_AR-T4 structures

**Figure 6:**
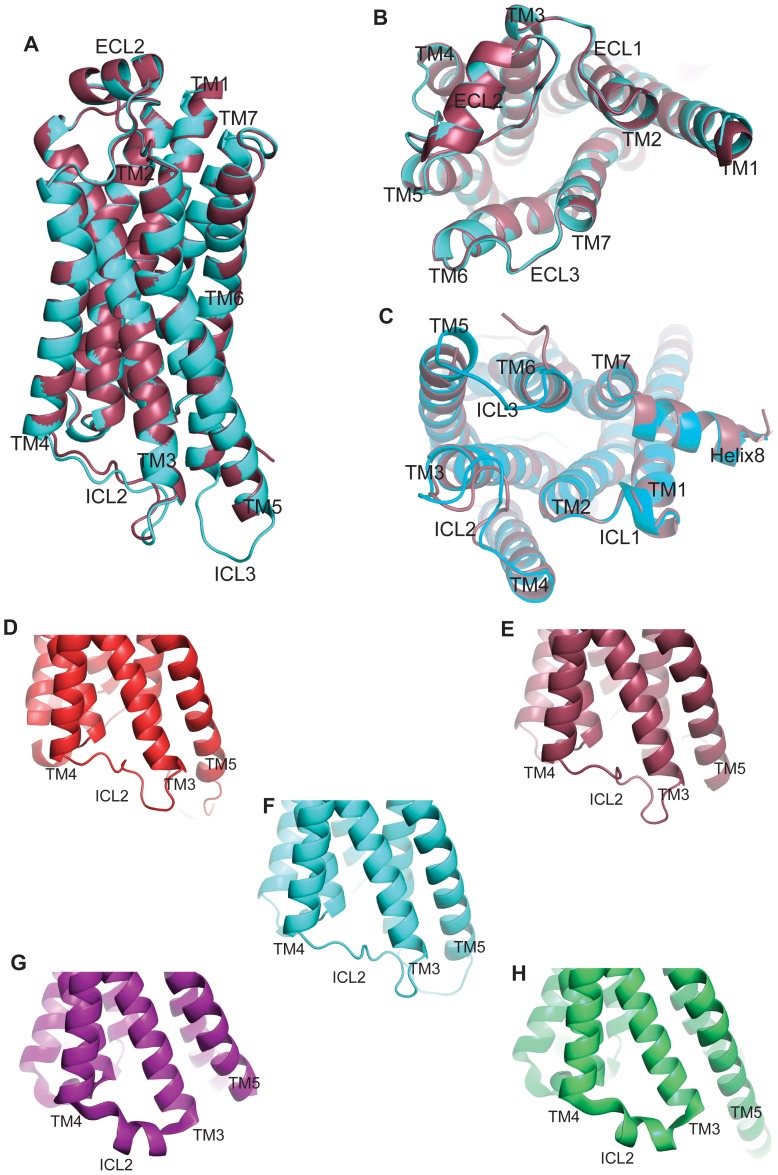
Structural comparison between T4L-β2AR-ΔICL3 and some other β_2_AR structures. **A.** The superimposed structures of the T4L-β_2_AR-ΔICL3 and the β_2_AR-T4L (pdb 2RH1). The T4L-β_2_AR-ΔICL3 is shown in cyan and the β_2_AR-T4L is shown in raspberry. **B.** The extracellular side view of the superposed structures. **C.** The intracellular side view of the superposed structures. **D.** ICL2 in the β_2_AR-Fab5 structure (pdb 2R4R). **E.** ICL2 in the β_2_AR-T4L structure (pdb 2RH1). **F.** ICL2 in the T4L-β_2_AR-ΔICL3 structure. **G.** ICL2 in the structure of β_2_AR stabilized by Nb80 (pdb 3P0G). **H.** ICL2 in the β_2_AR-Gs structure (pdb 3SN6).

The structures of the β_2_AR in T4L-β_2_AR-Δ-ICL3 (pdb 4GBR) and β_2_AR-T4L (pdb 2RH1) are very similar to each other (Fig. 6), with an overall root mean square deviation of 0.32 Å. The structures have similar solvent accessible surface areas: 25,000 Å^2^ for β_2_AR-T4L and 24000 Å^2^ for T4L-β_2_AR-Δ-ICL3. The slightly lower value for T4L-β_2_AR-Δ-ICL3 is due to more extensive packing interactions between T4L and the receptor. Only minor differences can be observed in these two structures, presumably due to different crystal packing patterns. The similarity of the structures determined independently through different strategies further validates the fusion protein approach, demonstrating that structural distortions due to protein engineering or crystal packing are unlikely.

Of interest, ICL2 in the two inactive structures of β_2_AR-Fab5 and β_2_AR-T4L is in an extended loop while it is an alpha helix in both active structures: the β_2_AR-Gs complex [11] and the β_2_AR stabilized by Nb80 [2]. In both of the inactive structures (β_2_AR-Fab5 and β_2_AR-T4L), ICL2 participates in lattice contacts that may influence its conformation. However, in the T4L-β_2_AR-Δ-ICL3 structure ICL2 is not involved in packing interactions, yet is an extended loop that is nearly identical to that observed in the other inactive state β_2_AR structures (Fig. 6). Thus, this extended loop structure may reflect an inactive state.

## Discussion

The majority part of a G-protein coupled receptor is surrounded by lipids or detergents, allowing very limited hydrophilic surface for crystal packing contacts. It has been shown that increasing the hydrophilic surface at the cytoplasmic side of the receptor can facilitate GPCR crystallization. However, insertion of T4L or binding of an antibody to ICL3 prevents GPCRs from forming signaling complexes with cytosolic protein partners. As an alternative strategy, we used an amino terminal T4L fusion to increase the extracellular hydrophilic surface available for forming crystal lattice contacts.

Our initial efforts to generate antibodies that recognize the extracellular surface of the β_2_AR were not successful. However, even if they were successful, these antibodies could not be used for other GPCRs. In contrast, the N-T4L fusion strategy may be more broadly applicable to other GPCRs and other membrane proteins. Our results demonstrate that the signal peptide used was sufficient to facilitate translocation of T4L domain across the endoplasmic reticulum membrane, ensuring proper orientation of TM1. Although it may compromise the rigidity of the fusion protein, a relatively flexible linker may be necessary to allow the receptor and the T4L to fold correctly. The optimal length of the linker between T4L and the amino terminus may differ for different GPCRs.

Compared with our previous strategies that utilized T4L or an antibody at the cytoplasmic surface, the N-terminal T4L fusion strategy allows for interactions between the β_2_AR and signaling and regulatory proteins as demonstrated by the recent β_2_AR-Gs complex structure. This approach also offers a protein engineering alternative for GPCRs and other membrane proteins that do not tolerate insertion of T4L or other hydrophilic proteins in cytoplasmic loops.

In conclusion, fusion of T4L to the amino terminus of a GPCR can facilitate crystallogenesis. This approach can also facilitate the formation of crystals of a GPCR in complex with a cytoplasmic signaling protein.

## Materials and Methods

### Generation of N-T4L fused β2AR constructs

The human β_2_AR in the pFastbac1 Sf9 expression vector truncated at amino acid 365 in the cytoplasmic tail (β_2_AR365) [1] was used as the starting template for generating the N-T4L fused β_2_AR constructs. The HA signal peptide followed by a FLAG epitope tag and a tobacco etch virus (TEV) protease recognition sequence were added to the N-terminus of the receptor to facilitate expression and purification. A point mutation of N187E was also introduced in the second extracellular loop to remove a glycosylation site (Fig. 1).

DNA cassettes encoding two different versions of T4L lysozyme (full length or with truncated C-terminus) with different numbers of additional alanines attached to the C-terminus were generated and amplified by PCR using the original β_2_AR-T4L [3] as the template and synthetic oligonucleotides as primers. These different cassettes were inserted into the β_2_AR365 construct between the end of the TEV protease recognition sequence and Asp29, Glu30 or Val31 of the receptor as shown in (Fig. 1) by using the Quickchange multi protocol (Stratagene). Two point mutations M96T, M98T were also introduced into the β_2_AR sequence. Residues from Ser235 to Lys263 in the third intracellular loop were deleted with the Quickchange multi protocol using synthetic oligonucleotides as mutation primers. All the constructs were confirmed by DNA sequencing. The protein sequence of T4L-β_2_AR-Δ-ICL3 is shown below:

*MKTIIALSYIFCLVFA* DYKDDDDAENLYFQ*GNIFEMLRIDEGLRLKIYKDTEGYYTIGIGHLLTKSPSLNAAKSELDKAIGRNTNGVITKDEAEKLFNQDVDAAVRGILRNAKLKPVYDSLDAVRRAALINMVFQMGETGVAGFTNSLRMLQQKRWDEAAVNLAKSRWYNQTPNRAKRVITTFRTGTWDAYAADEVWVVGMGIVMSLIVLAIVFGNVLVITAIAKFERLQTVTNYFITSLACADLVMGLAVVPFGAAHILTKTWTFGNFWCEFWTSIDVLCVTASIETLCVIAVDRYFAITSPFKYQSLLTKNKARVIILMVWIVSGLTSFLPIQMHWYRATHQEAINCYAEETCCDFFTNQAYAIASSIVSFYVPLVIMVFVYSRVFQEAKRQLQKIDKFCLKEHKALKTLGIIMGTFTLCWLPFFIVNIVHVIQDNLIRKEVYILLNWIGYVNSGFNPLIYCRSPDFRIAFQELLCLRRSSLKAYGNGYSSNGNTGEQSG

(the HA signal peptide is shown in italic letters; the FLAG epitope tag is shown in letters with underscore; the TEV recognition sequence is marked with a box and the cleavage site is shown with an asterisk; the full length T4L is shown in orange; the β_2_AR sequence from Asp29 to Gly365 excluding Ser235 to K263 is shown in cyan, the 2-Ala linker is shown in blue).

The entire T4L-β_2_AR-Δ-ICL3 gene described above was further cloned into the Best-Bac Sf9 expression vector pvl1393 (Expression Systems, Woodland, CA) using the restriction enzyme digestion site XbaI and EcoRI. This version of T4L-β_2_AR-Δ-ICL3 construct was also confirmed by DNA sequencing.

### Whole cell binding to assess the expression yield of each construct

Recombinant baculovirus was made from the pFastbac1 Sf9 expression vector for each of the constructs illustrated in Fig. 1 using the Invitrogen protocol. Sf9 cells (Expression Systems, Woodland, CA) at a density of 4 million/ml were infected with second passage virus at different ratios of virus stock to cell culture (1∶20, 1∶50, and 1∶100). After 48 hours, 5 µl of the infected cells were incubated with 10 nM of [^3^H]-dihydroalprenolol (DHA) in 500 µl of binding buffer (75 mM Tris, 12.5 mM MgCl2, 1 mM EDTA, pH 7.4, supplemented with 5 mg/ml BSA). Cells were harvested and washed with cold binding buffer using a Brandel harvester. Bound [^3^H]DHA was measured with a scintillation counter (Beckman). Non-specific binding of [^3^H]DHA was assessed by including 10 µM of alprenolol (Sigma) in the same binding reaction. The expression level of each construct was determined using the specific activity of the bound [^3^H]DHA. Each experiment was performed in triplicate.

### Saturation and competition binding assays

Membranes from Sf9 cells expressing either wild-type β2AR or T4L-β_2_AR-Δ-ICL3 were prepared based on a previously described protocol [12]. In each reaction in the saturation binding assay, membranes containing approximately 0.2 pmol receptor were incubated with concentrations of [^3^H]DHA ranging from 5pM to 10 nM in 500 µl of buffer (75 mM Tris, 12.5 mM MgCl_2_, 1 mM EDTA, pH 7.4, supplemented with 0.5 mg/ml BSA) at room temperature with shaking at 230 rpm for 1 hour. Membranes were isolated from free [^3^H]DHA using a Brandel harvester and washed three times with cold buffer. The amount of receptor bound [^3^H]DHA was measured using a scintillation counter (Beckman). Non-specific binding of the [^3^H]DHA in each reaction was assessed by including 1 µM alprenolol (Sigma) in the same reaction. In each reaction for the competition binding assay, membrane containing approximately 0.2 pmol receptor was incubated with 1 nM [^3^H]DHA and different concentrations of (−)-isoproterenol (Sigma) ranging from 1 nM to 1 mM. Membranes were harvested and washed three times with cold buffer. The bound [^3^H]DHA was counted as described above. Non-specific [^3^H]DHA was assessed by replacing (−)-isoproterenol with 1 µM alprenolol. All the binding data was analyzed by non-linear regression method using Graphpad Prism. Each experiment was performed in triplicate.

### GTPγS binding assay

The T4L-β_2_AR-Δ-ICL3 or the wild type β_2_AR was reconstituted in HDL particles (receptor·rHDL) as described by Whorton *et al* [13]. The Gs heterotrimer (Gαs, his6-β1, γ2) was expressed in Sf9 cells and purified as described by Kozasa *et al* [14]. In order to obtain reconstituted Gs-receptor·rHDL complex, the purified Gs was added into preformed receptor·rHDL at a molar ratio of 10∶1 (the concentration of the Gs stock was high such that the contained detergent was diluted for about 1000 fold to a concentration well below its critical micelle concentration). In each GTPγS binding reaction, the above described Gs-receptor·rHDL mixture was preincubated with different ligands (no ligand, 10 µM of ICI-118551, or 10 µM of isoproterenol, respectively; the final concentration of the Gs is 1 nM and the receptor·rHDL is 0.1 nM) in 500 µl of buffer (75 mM Tris, 12.5 mM MgCl_2_, 1 mM EDTA, pH 7.4, supplemented with 0.5 mg/ml BSA) for 10 min at room temperature followed by the addition of 1 nM [^35^S]GTPγS (Perkin Elmer Life Sciences). After 20 min of shaking at room temperature, the reconstituted Gs-receptor·rHDL mixture was isolated from free [^35^S]GTPγS using a Brandel harvester and washed three times with cold buffer. The amount of bound [^35^S]GTPγS was measured using a scintillation counter. Non-specific binding of the [^35^S]GTPγS was assessed by including 10 µM of cold GTPγS (Sigma). Data from three repeated experiments was analyzed using Graphpad Prism. Each experiment was performed in triplicate.

### Expression and purification of T4L-β_2_AR-Δ-ICL3 from baculovirus-infected Sf9 cells

Recombinant baculovirus was made from pvl1393-T4L-β_2_AR-Δ-ICL3 using Best-Bac expression system, as described by the system protocol (Expression Systems). T4L-β_2_AR-Δ-ICL3 was expressed by infecting Sf9 cells at a density of 4 million/ml with a second passage baculovirus stock using 1 ml of virus stock per 50 ml of cell culture. 1 µM of the antagonist alprenolol was included to enhance the receptor stability and yield. The infected cells were harvested after 48 hs of incubation at 27°C.

Cell pellets were lysed by vigorous stirring in lysis buffer (10 mM TRIS-Cl pH 7.5, 2 mM EDTA, 10 ml of buffer per gram of cell pellet) supplemented with protease inhibitor Leupeptin (2.5 µg/ml final concentration, Sigma) and Benzamindine (160 µg/ml final concentration, Sigma) for 15 minutes. The T4L-β_2_AR-Δ-ICL3 protein was extracted from the cell membrane by dounce homogenization in solubilization buffer (100 mM NaCl, 20 mM TRIS-Cl, pH 7.5, 1% Dodecylmaltoside) supplemented with Leupeptin and Benzamindine (2.5 µg/ml and 160 µg/ml final concentration, respectively). 10 ml of solubilization buffer was used for each gram of cell pellet. The Dodecylmaltoside (DDM)-solubilized T4L-β_2_AR-Δ-ICL3 bearing the FLAG epitope was then purified by M1 antibody affinity chromatography (Sigma). Extensive washing using HLS buffer (100 mM NaCl, 20 mM HEPES pH 7.5, 0.1%DDM) was performed to remove alprenolol. The protein was then eluted with HLS buffer containing a saturating concentration of cholesterol hemisuccinate (CHS) and supplemented with 5 mM EDTA and 200 µg/ml free FLAG peptide. The HLS-CHS buffer was prepared by mixing HLS with 0.05% (weight:volume) CHS for 1 hr at room followed by filtration through a 0.2 µ filter to remove undissolved CHS.

The eluted T4L-β_2_AR-Δ-ICL3 was further purified by affinity chromatography using alprenolol-Sepharose as previously described [3] in order to isolate functional T4L-β_2_AR-Δ-ICL3 from non-functional protein. HHS buffer (350 mM NaCl, 20 mM HEPES pH 7.5, 0.1%DDM) supplemented with 300 µM alprenolol and a saturating concentration of CHS (prepared as above) was used to elute the protein. The eluted T4L-β_2_AR-Δ-ICL3 bound with alprenolol was then re-applied to M1 resin, allowing exchange of alprenolol with carazolol in HHS buffer supplemented with 30 nM carazolol. T4L-β_2_AR-Δ-ICL3 bound with carazolol was then eluted from M1 resin with HHS buffer supplemented with 5 mM EDTA, 200 µg/ml free FLAG peptide and saturating concentration of CHS (prepared as described above). The FLAG epitope tag of T4L-β_2_AR-Δ-ICL3 was removed by the treatment of tobacco etch virus (TEV) protease (Invitrogen) for 3 h at room temperature or overnight at 4°C. The untagged T4L-β_2_AR-Δ-ICL3-cazazolol complex was then further purified by size-exclusion chromatography (SEC) using an S200 column (GE healthcare) equilibrated in 100 mM NaCl, 10 mM HEPES pH 7.5, 0.1% DDM and 1 nM carazolol. The same buffer was used as the running buffer for SEC. The purity of the final T4L-β_2_AR-ΔICL3 is better than 90%, as assessed by SDS-PAGE.

### Crystallization of the T4L-β_2_AR-ΔICL3-carazolol complex

The purified T4L-β_2_AR-Δ-ICL3-carazolol complex was concentrated to a final concentration of 60 mg/ml using centricon Vivaspin (GE healthcare). The complex was crystallized using the lipid cubic phase (LCP) method as previously described [8]. The protein complex was mixed with lipid moloolein with a 1∶1.5 mass ratio at room temperature. 0.03 µl of the protein-lipid mixture drop was deposited in each well of a 96-well glass sandwich plate (Molecular Dimensions). The drop was then overlaid with 0.65 µl of precipitant and the well was sealed by glass coverslip. By using this method, the T4L-β_2_AR-Δ-ICL3-carazolol complex was crystallized in 37% PEG300 (v/v), 0.1 M Bis-Tris propane, pH 6.5, 0.1 M ammonium phosphate after 2 days of incubation in 20°C.

### Data collection and structure determination

Crystals were harvested and frozen in liquid nitrogen directly without using additional cryo-protectant. Diffraction data from 15 different crystals were measured using the GM/CA-CAT minibeam at 23-ID-D, Advance Photon Source, Argonne National Labs. The data were processed with HKL2000 [15] and the structure solved by molecular replacement using Molrep. Further model rebuilding was performed by using Coot [16] and the structure was refined with Phenix [17]. The validation of the final structural model was performed using Molprobity [18]. Data processing and refinement statistics are shown in Table 1. The root mean square deviaion value of 0.32 was calculated using pymol. The cutoff value is 3 and 268 out of 282 Cα atoms of the two structures (4GBR and 2RH1) were included in the structural alignment. Solvent accessible surface area calculation was also performed using Pymol.

## References

[1] Rasmussen SG, Choi HJ, Rosenbaum DM, Kobilka TS, Thian FS, et al (2007) Crystal structure of the human beta2 adrenergic G-protein-coupled receptor. Nature 450: 383–387. 17952055 10.1038/nature06325

[2] Rasmussen SG, Choi HJ, Fung JJ, Pardon E, Casarosa P, et al (2011) Structure of a nanobody-stabilized active state of the beta(2) adrenoceptor. Nature 469: 175–180. 21228869 10.1038/nature09648 PMC3058308

[3] Rosenbaum DM, Cherezov V, Hanson MA, Rasmussen SG, Thian FS, et al (2007) GPCR engineering yields high-resolution structural insights into beta2-adrenergic receptor function. Science 318: 1266–1273. 17962519 10.1126/science.1150609

[4] Jaakola VP, Griffith MT, Hanson MA, Cherezov V, Chien EY, et al (2008) The 2.6 Angstrom Crystal Structure of a Human A2A Adenosine Receptor Bound to an Antagonist. Science 322: 1211–1217. 18832607 10.1126/science.1164772 PMC2586971

[5] Chien EY, Liu W, Zhao Q, Katritch V, Han GW, et al (2010) Structure of the human dopamine D3 receptor in complex with a D2/D3 selective antagonist. Science 330: 1091–1095. 21097933 10.1126/science.1197410 PMC3058422

[6] Wu B, Chien EY, Mol CD, Fenalti G, Liu W, et al (2010) Structures of the CXCR4 Chemokine GPCR with Small-Molecule and Cyclic Peptide Antagonists. Science 330: 1066–1071. 20929726 10.1126/science.1194396 PMC3074590

[7] Shimamura T, Shiroishi M, Weyand S, Tsujimoto H, Winter G, et al (2011) Structure of the human histamine H1 receptor complex with doxepin. Nature 475: 65–70. 21697825 10.1038/nature10236 PMC3131495

[8] Kruse AC, Hu J, Pan AC, Arlow DH, Rosenbaum DM, et al (2012) Structure and dynamics of the M3 muscarinic acetylcholine receptor. Nature 482: 552–556. 22358844 10.1038/nature10867 PMC3529910

[9] Haga K, Kruse AC, Asada H, Yurugi-Kobayashi T, Shiroishi M, et al (2012) Structure of the human M2 muscarinic acetylcholine receptor bound to an antagonist. Nature 482: 547–551. 22278061 10.1038/nature10753 PMC3345277

[10] Hanson MA, Roth CB, Jo E, Griffith MT, Scott FL, et al (2012) Crystal structure of a lipid G protein-coupled receptor. Science 335: 851–855. 22344443 10.1126/science.1215904 PMC3338336

[11] Rasmussen SG, DeVree BT, Zou Y, Kruse AC, Chung KY, et al (2011) Crystal structure of the beta2 adrenergic receptor-Gs protein complex. Nature 477: 549–555. 21772288 10.1038/nature10361 PMC3184188

[12] Swaminath G, Steenhuis J, Kobilka B, Lee TW (2002) Allosteric modulation of beta2-adrenergic receptor by Zn(2+). Mol Pharmacol 61: 65–72. 11752207 10.1124/mol.61.1.65

[13] Whorton MR, Bokoch MP, Rasmussen SG, Huang B, Zare RN, et al (2007) A monomeric G protein-coupled receptor isolated in a high-density lipoprotein particle efficiently activates its G protein. Proc Natl Acad Sci 104: 7682–7687. 17452637 10.1073/pnas.0611448104 PMC1863461

[14] Kozasa T, Gilman AG (1995) Purification of recombinant G proteins from Sf9 cells by hexahistidine tagging of associated subunits. Characterization of alpha 12 and inhibition of adenylyl cyclase by alpha z. J Biol Chem 270: 1734–1741. 7829508 10.1074/jbc.270.4.1734

[15] Otwinowski Z, Minor W (1997) Processing of x-ray diffraction data collected in oscillation mode. Methods Enzymol 276: 307–326. 10.1016/S0076-6879(97)76066-X 27754618

[16] Emsley P, Cowtan K (2004) Coot: model-building tools for molecular graphics. Acta Crystallogr D Biol Crystallogr 60: 2126–2132. 15572765 10.1107/S0907444904019158

[17] Afonine PV, Grosse-Kunstleve RW, Adams PD (2005) A robust bulk-solvent correction and anisotropic scaling procedure. Acta Crystallogr D Biol Crystallogr 61: 850–855. 15983406 10.1107/S0907444905007894 PMC2808320

[18] Chen VB, Arendall WB 3rd, Headd JJ, Keedy DA, Immormino RM, et al (2010) MolProbity: all-atom structure validation for macromolecular crystallography. Acta Crystallogr D Biol Crystallogr 66: 12–21. 20057044 10.1107/S0907444909042073 PMC2803126

